# Simulation-based Training in Ectopic Pregnancy and Salpingostomy

**DOI:** 10.7759/cureus.5116

**Published:** 2019-07-10

**Authors:** Isabella A Sabatina, Jheel V Shah, David Gothard, Derek A Ballas

**Affiliations:** 1 Obstetrics and Gynecology, Northeast Ohio Medical University (NEOMED), Akron, USA; 2 Obstetrics and Gynecology, Summa Health System, Akron, USA; 3 Clinical Research, Summa Health System, Akron, USA

**Keywords:** salpingectomy, salpingostomy, ectopic, pregnancy, simulation, medical education, obstetrics and gynecology

## Abstract

Objective

Ectopic pregnancy leads to approximately 3% of deaths in pregnancy. Surgical management is indicated when patients are hemodynamically unstable or have signs of a ruptured ectopic pregnancy. Salpingectomy is more commonly performed, but salpingostomy is preferred in a patient with prior salpingectomy with a desire for future pregnancy. Due to the lack of exposure, salpingostomy is not frequently performed and most residents do not feel adequately trained. Our goal was to provide a hands-on simulation about ectopic pregnancy and salpingostomy in hopes that the simulation will improve the resident’s confidence and knowledge in recognizing an ectopic pregnancy, identifying an appropriate candidate for surgical management, and performing a salpingostomy.

Methods

The educational initiative was aimed towards postgraduate year (PGY) 1-4 OB/GYN residents (n=11). Knowledge and confidence questionnaires were given to participants prior to and post-simulation. A gynecologic mannequin was modified by taking the existing pelvic organs and creating a tubal pregnancy. In the first part of the simulation, a hemodynamically unstable patient presented with lab and imaging findings consistent with an ectopic pregnancy. Once recognized and the decision made for surgical intervention, participants were transferred to a simulated operating room where they performed salpingostomy or salpingectomy on the mannequin. The simulation was followed by a debriefing session to discuss the actions and thought processes of participants, provide reflection, and incorporate improvement opportunities for future cases. Finally, participants engaged in a didactic lecture where they were educated about the incidence, presentation, and management of tubal ectopic pregnancy.

Results

Analysis of the knowledge questionnaires showed the median score pre- and post-intervention was 9 and 12, respectively, with a median change of 3 (p=0.001). The median confidence value pre- and post-intervention were 28 and 42, respectively, with a median value change of 12 (p<0.001).

Conclusion

Our intervention improved residents' confidence and knowledge in recognizing an ectopic pregnancy, identifying an appropriate candidate for surgical management, and performing a salpingostomy.

## Introduction

Ectopic pregnancy has been increasing in frequency worldwide, becoming a leading cause of morbidity and mortality in pregnancy [1]. It is thought to be as common as 1% to 2% of all pregnancies and contributes to 2.7% of pregnancy-related deaths [2-3]. Ectopic pregnancies can be treated with various medical and surgical interventions, including expectant management, methotrexate, salpingostomy, and salpingectomy. Management decision should be guided by patient-informed preference, clinical evaluation, and laboratory and radiological information [4]. Surgical management is necessary if patients have an absolute contraindication to methotrexate use, are hemodynamically unstable, have symptoms of a ruptured ectopic pregnancy, or have signs of intraperitoneal bleeding [4]. According to randomized control trials, there is no fertility advantage of salpingostomy versus salpingectomy in the management of tubal ectopic pregnancy with a healthy contralateral tube [5]. However, if there is a desire for future fertility with a diseased or absent contralateral tube, salpingostomy is the preferred surgical intervention unless contraindicated [1]. Salpingectomy is generally required when there is extensive tubal damage or bleeding [1].

There is controversy surrounding the surgical management of tubal ectopic pregnancy, with salpingectomy often times being favored over salpingostomy. The risk of retained trophoblastic material is much higher with a salpingostomy as compared to a salpingectomy, which leads to more follow-up and possible intervention [5-6]. Salpingostomy follow-up includes weekly β-hCG levels until levels are undetectable and may also require treatment with methotrexate if β-hCG levels are persistently elevated, suggesting the presence of retained trophoblastic material [7-8]. Another reason salpingectomy is favored is due to physician experience with each procedure. Salpingectomy is routinely performed for ectopic pregnancies, permanent sterilization, ovarian cancer prevention, and concomitantly with hysterectomies [9]. However, indications for salpingostomy are only for ectopic pregnancy and to recanalize a previously damaged fallopian tube (hydrosalpinx, adhesions) [10]. The former indications for surgery are more common than the latter, making it unlikely that physicians will have encountered or have been properly instructed in performing a salpingostomy.

Although laparoscopy has become more widespread, there is often limited basic laparoscopic and hysteroscopic simulation teaching during residency [11]. In order to increase resident performance and decrease procedural complications, there must be an increase in exposure to techniques not routinely performed during residency. Due to the relative rarity of performing a salpingostomy, simulation training is necessary to provide physician exposure to the procedure. The use of simulation-based training has been widely supported as a pedagogic tool to improve both physician skills and patient outcomes [12-13]. There has also been an emphasis on incorporating simulations into physician training, especially for less frequently encountered patient scenarios [13]. A recent review of the literature identifies a scarcity of educational materials in performing salpingostomy for ectopic pregnancy. The purpose of this simulation is to provide physicians the opportunity to identify an ectopic pregnancy, know the indications for surgical management, and practice the steps to performing a salpingostomy.

## Materials and methods

**Figure 1:**
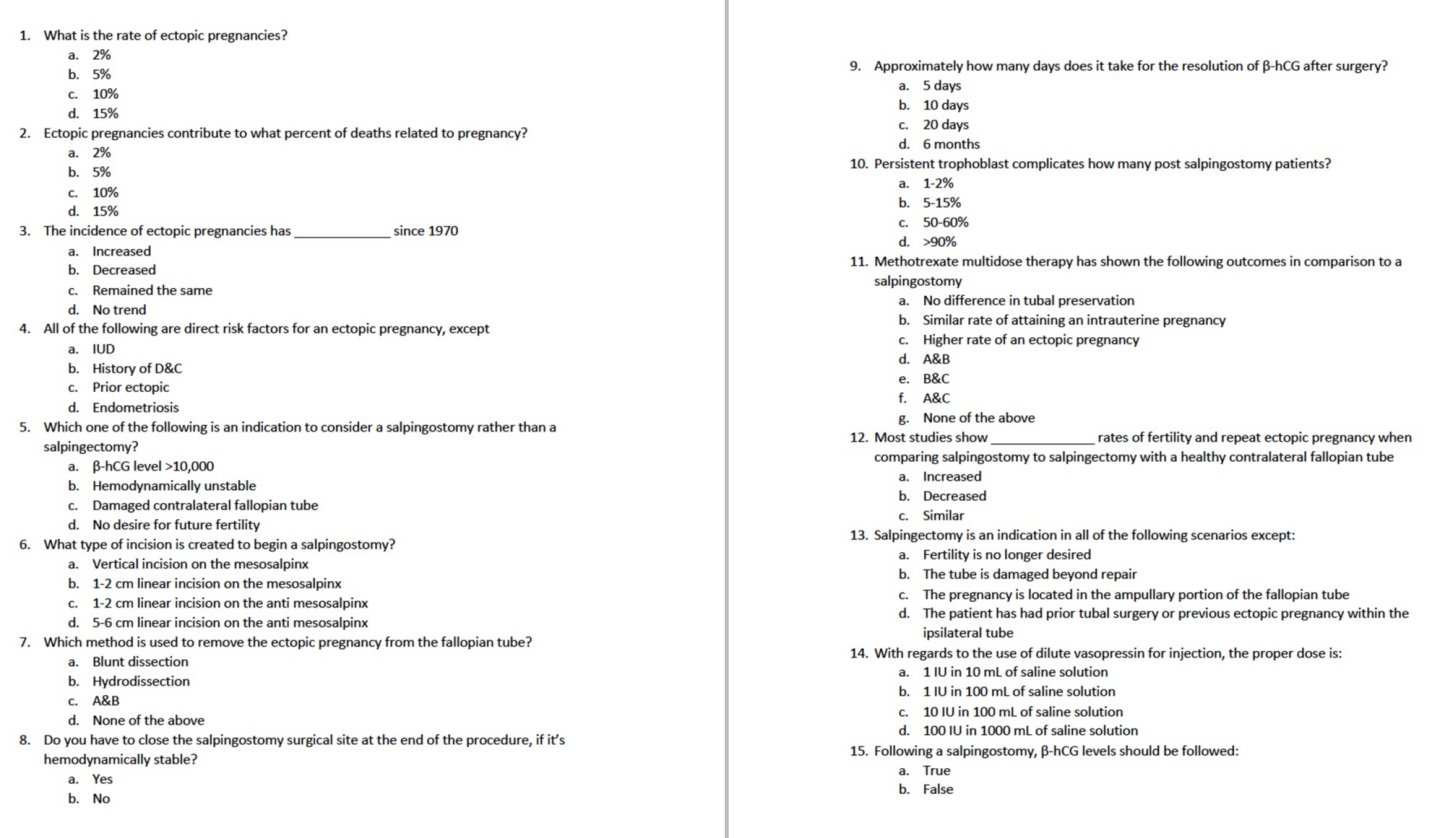
Knowledge questionnaire distributed pre- and post-simulation

**Figure 2:**
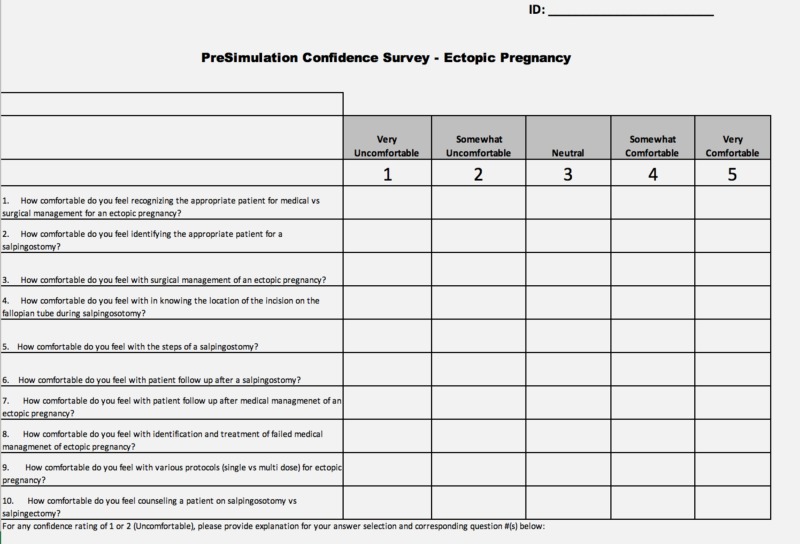
Confidence questionnaire distributed pre- and post-simulation

This quasi-experimental study was conducted in a simulation lab at a university-affiliated community hospital. It was submitted to the institutional review board and qualified for exempt status as an educational intervention. Eleven postgraduate year (PGY) 1 to PGY 4 obstetrics and gynecology resident physicians at Akron City Hospital voluntarily participated in a written pre-intervention knowledge and confidence assessment immediately prior to the intervention in the simulation lab (Figures 1-2). The components of the study design are outlined in Table 1.

**Table 1:** Outline of study design

Pre-Intervention	Intervention				Post Intervention
Knowledge and Confidence Questionnaire	Simulation – identifying a candidate for surgical management of an ectopic pregnancy	Simulation – performing a salpingostomy	Debriefing Session	Didactic Session	Knowledge and Confidence Questionnaire

**Figure 3:**
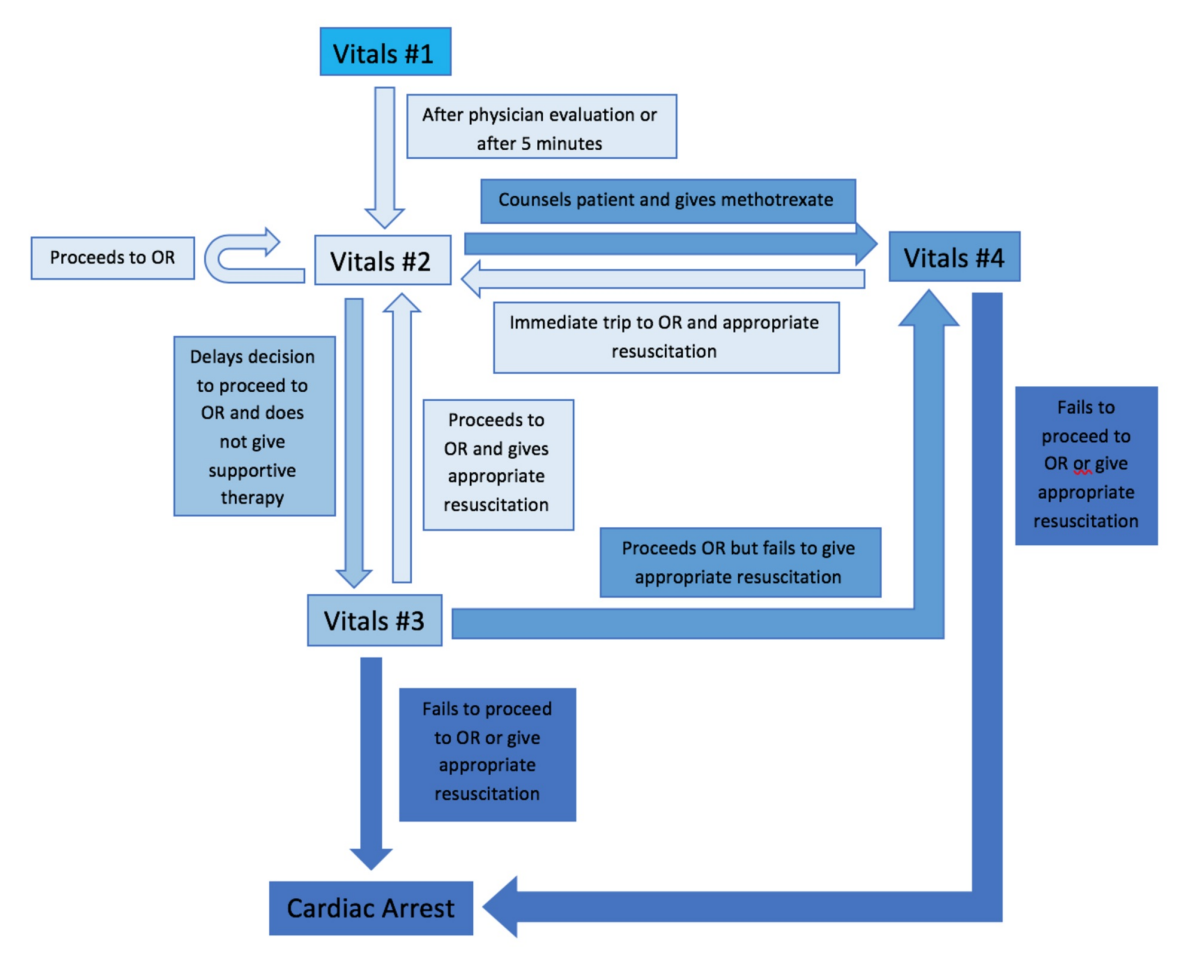
Flow chart of patient condition during simulation progression

In the first part of the simulation, the residents were presented with a case of a hemodynamically unstable patient, along with lab and imaging findings upon request consistent with an ectopic pregnancy. The patient’s vital signs (Table 2) and lab findings worsened if the residents failed to intervene appropriately (Figure 3).

**Table 2:** Vital signs for simulation

Vital Signs	Heart Rate (beats per minute)	Blood Pressure (mmHg)	Temperature (Fahrenheit)	Oxygen Saturation (%)	Respiratory Rate (breaths per minute)	Appearance
Vitals #1	112	95/65	98.6	98%	24	Appears comfortable and in no distress
Vitals #2	120	90/54	98.6	94%	26	Increasing abdominal pain
Vitals #3	130	80/50	98.6	91%	26	Extreme agitation and pain
Vitals #4	130	70/30	98.6	88%	18	Loses consciousness

**Figure 4:**
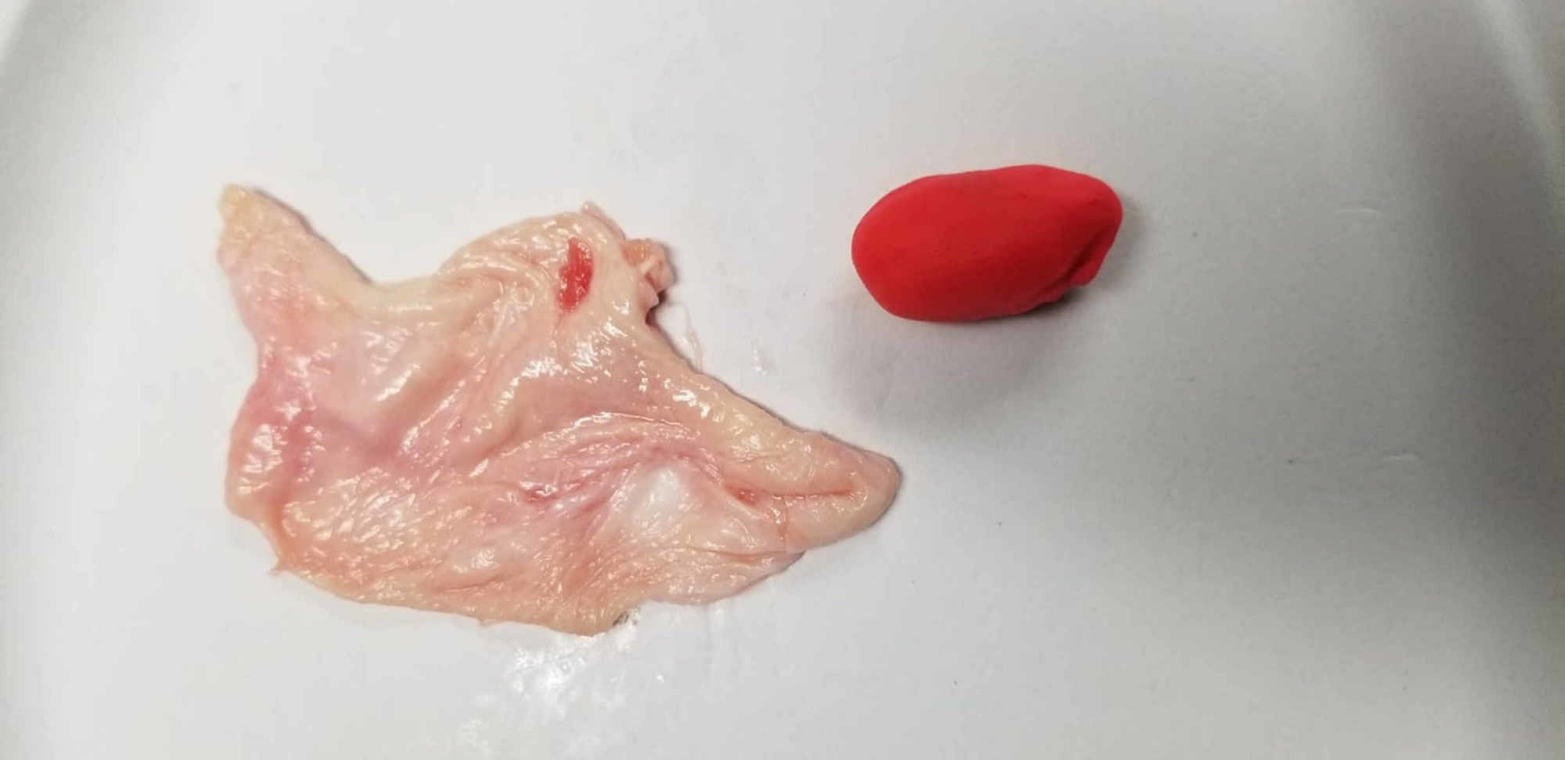
Chicken skin and Play-Doh used to simulate tubal ectopic pregnancy

**Figure 5:**
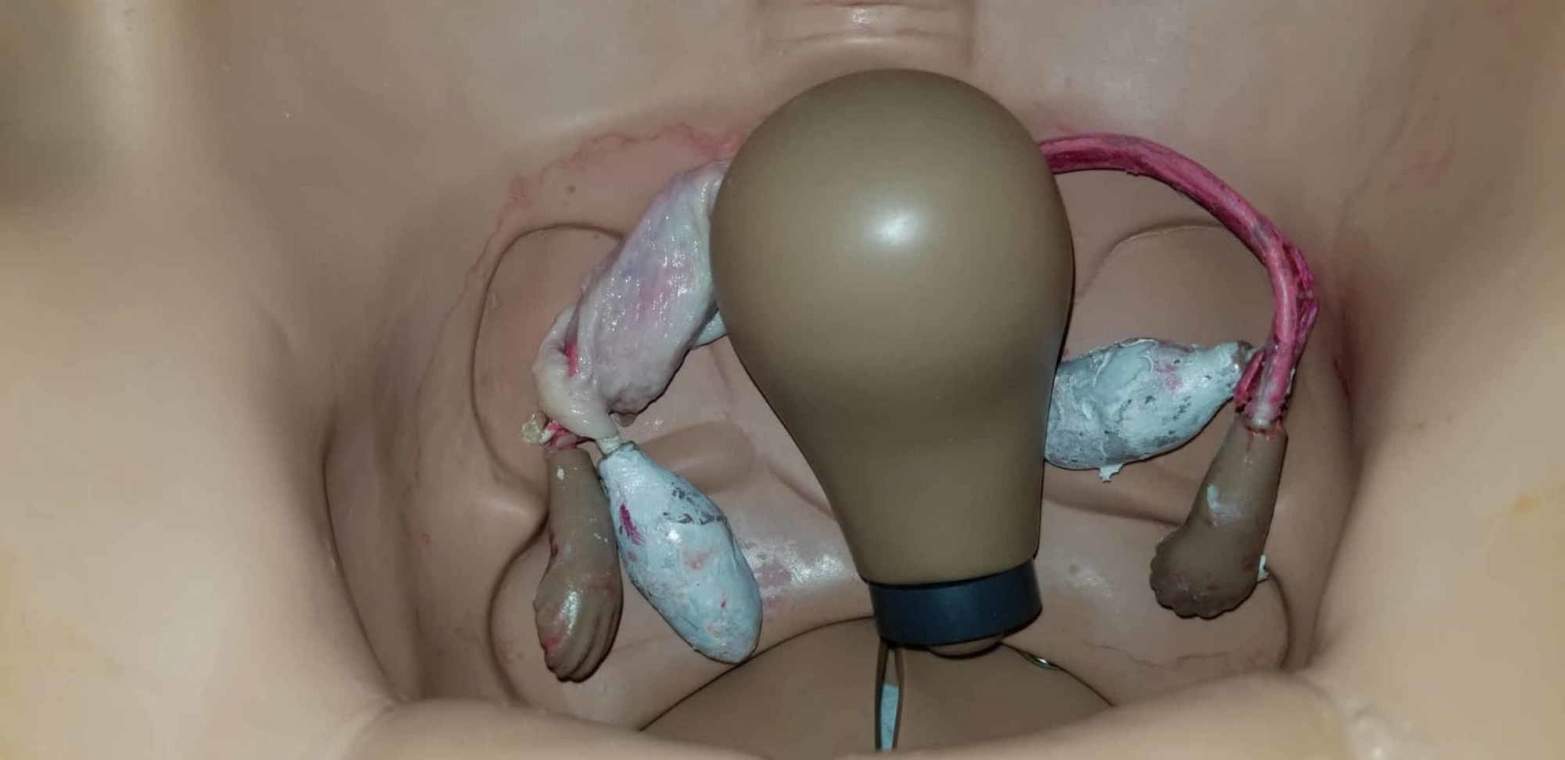
Mannequin with tubal ectopic pregnancy

**Figure 6:**
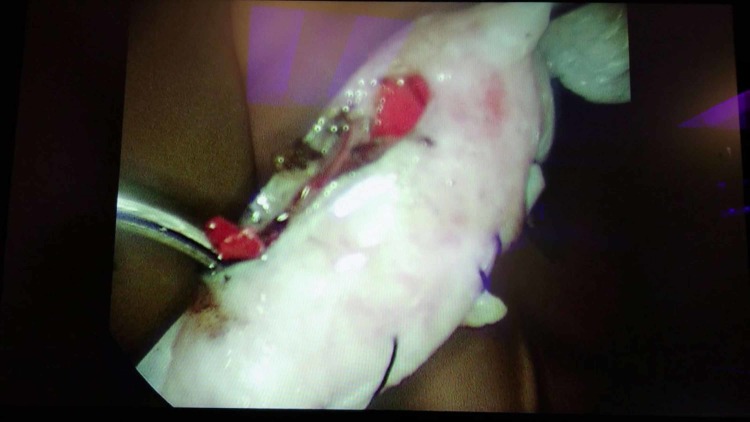
Laparoscopic image of the simulated ectopic pregnancy after electrocautery was used

Once recognized and the decision was made for surgical intervention, the participants were transferred to an operating suite within the simulation lab where they performed laparoscopic salpingostomy or salpingectomy on a mannequin. A gynecologic mannequin was modified by taking the model’s existing pelvic organs and creating a tubal pregnancy using chicken skin rolled into a tubular lumen with a dilation created by red Play-Doh embedded within the chicken skin, as seen in Figures 4-5. This enabled the residents to utilize electrocautery during their surgical simulation (Figure 6). The simulation was considered over when the ectopic pregnancy was evacuated using either surgical approach.

The simulation was followed by a debriefing session to discuss the actions and thought processes of the participants, provide reflection, and incorporate improvement opportunities for future cases.

Finally, participants engaged in a didactic lecture where they were educated about the incidence, presentation, and management of a tubal ectopic pregnancy using a Microsoft PowerPoint presentation. Post-intervention knowledge and confidence assessment questionnaires were re-administered immediately after the didactic session in the simulation lab.

## Results

**Figure 7:**
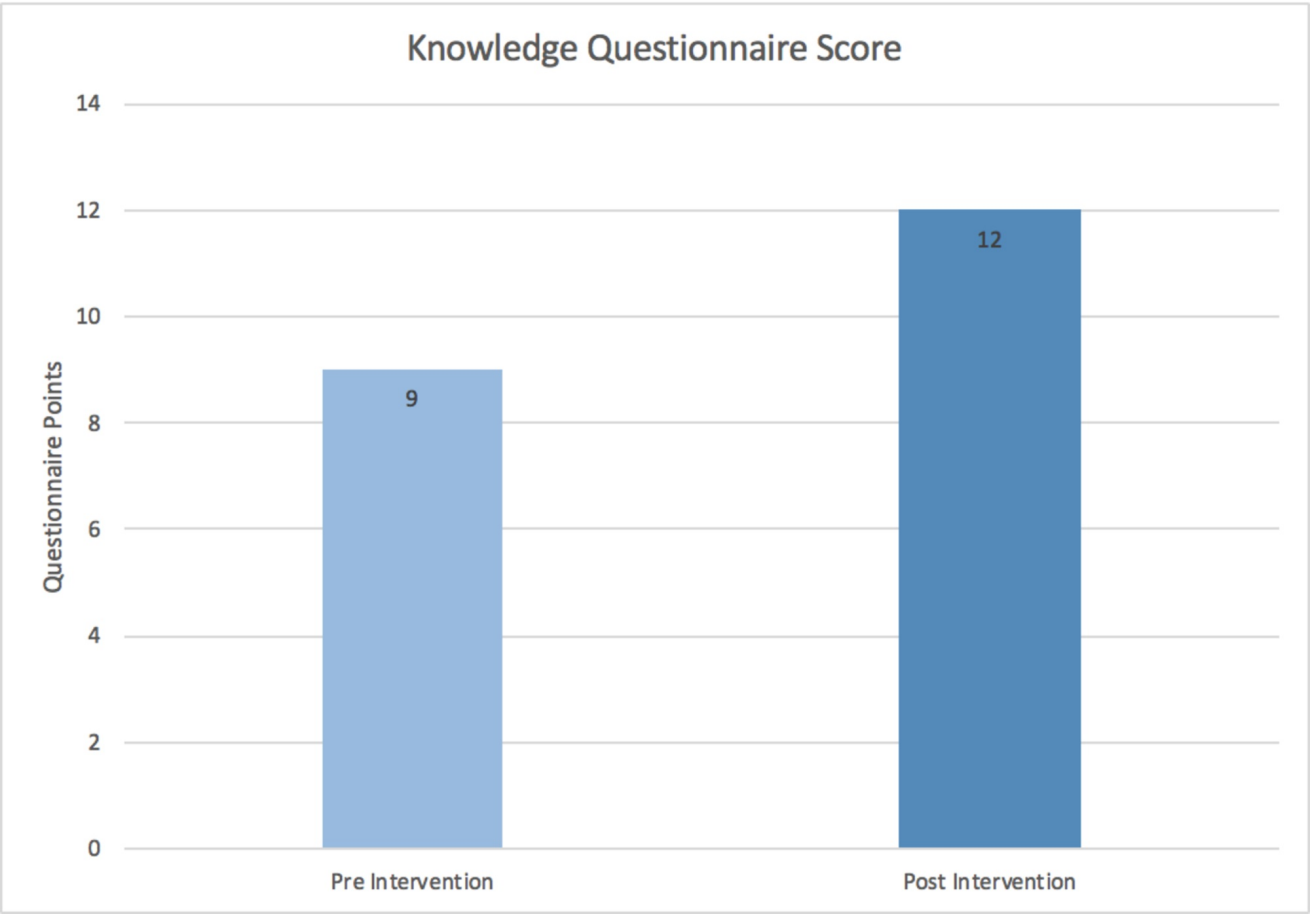
Knowledge questionnaire score

**Figure 8:**
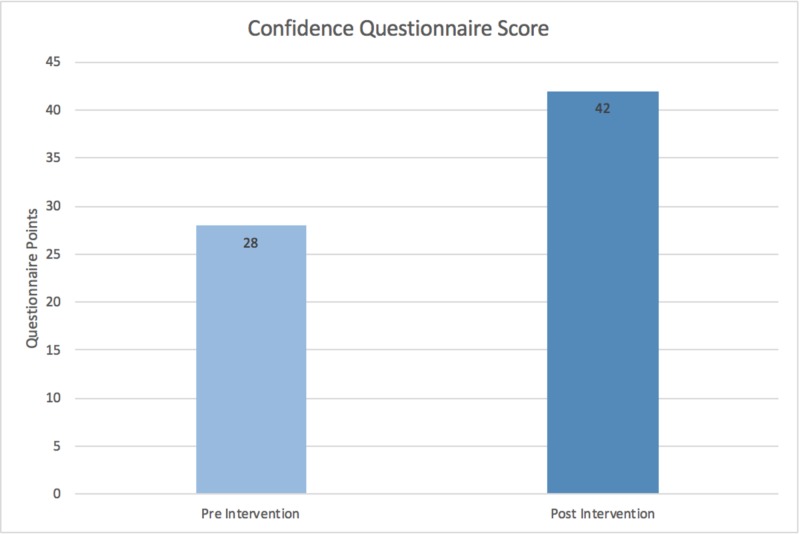
Confidence questionnaire score

The Wilcoxon signed rank test was used to evaluate statistical significance for both the knowledge and confidence values. The knowledge questionnaire was scored out of 15. With a median change of 3 (p=0.001), the pre- and post-intervention scores were 9 and 12, respectively (Figure 7). The median confidence questionnaire score pre- and post-intervention were 28 and 42 out of 75, respectively, with a median value change of 12 (p<0.001) (Figure 8).

## Discussion

Our educational initiative demonstrates a statistically significant increase in residents’ knowledge and confidence in recognizing and performing a salpingostomy after the simulation. This increase is most evident in the results of the questionnaire for the following: recognizing the appropriate patient for surgical management, the steps and follow-up of salpingostomy, and counseling a patient regarding salpingostomy versus salpingectomy.

One of the strengths of our study is its relevance to the practice scope of OB/GYN physicians. It provides exposure to a less frequently performed procedure that is well within the scope of practice of an OB/GYN. The simulation’s use of active participation rather than passive learning gives residents the opportunity for deliberate practice in a controlled environment. Participants in this training event had the unique opportunity to use electrocautery due to the use of chicken skin in the ectopic pregnancy model. Lastly, the minimal personnel and materials necessary for the simulation make this study reproducible in a typical hospital setting.

One of the limitations of our study includes a small sample size. Only 11 residents from a single institution participated in this study, which is not a generalizable representation of nationwide OB/GYN residents. Another limitation is the narrow timeframe of the study. Pre-intervention, intervention, and post-intervention activities all occurred in a single day. The spacing effect of learning supports that learning is greater when spread out over time as opposed to in a single session [14]. The study also only evaluated immediate changes in knowledge and confidence without the long-term assessment of knowledge retention. Another limitation is the lack of objective evaluation of participants’ skills and the significance of clinical application.

In the future, similar studies can be improved by increasing the time between pre-intervention, intervention, and post-intervention in order to emphasize the space effect on recall. It also would be beneficial to have long-term follow-up to evaluate knowledge and confidence retention. An objective analysis of procedural skills pre- and post-intervention would also be advantageous over our subjective analysis. This study could also be taken one step further to evaluate the simulation’s clinical impact on salpingostomy technique over a prolonged period of time.

## Conclusions

This study emphasizes the importance of identifying and appropriately managing an ectopic pregnancy with salpingostomy. Participants learn how to properly educate, accurately identify, and appropriately manage patients who would benefit from a salpingostomy. This study also shows the benefit of using simulation to improve both physician knowledge and confidence regarding salpingostomy.
